# The role and mechanism of chaperones Calnexin/Calreticulin in which ALLN selectively rescues the trafficking defective of HERG-A561V mutation

**DOI:** 10.1042/BSR20171269

**Published:** 2018-09-07

**Authors:** Ying Wang, Tingting Shen, Peiliang Fang, Junbo Zhou, Kenan Lou, Zemin Cen, Hai Qian, Jianqing Zhou, Ningsheng Liu, Jiangfang Lian

**Affiliations:** 1 Department of Cardiovascular, Ningbo Medical Center Lihuili Eastern Hospital, Ningbo, China; 2 Department of Cardiovascular, Taipei Medical University Ningbo Medical Center, Ningbo, China; 3 Department of Pathology, Nanjing Medical University, Nanjing, China; 4 Department of Cardiovascular, Ningbo City Medical Treatment Center Lihuili Hospital, Ningbo, China

**Keywords:** ALLN, chaperones Calnexin/Calreticulin, HERG-A561V mutation

## Abstract

Long QT (LQT) type 2 (LQT2) is caused by HERG mutation. L539fs/47 encodes a truncated protein, and its mechanisms in HERG mutation are unknown. HERG mutation plasmids were overexpressed in HEK293T cells, respectively, followed by analyzing lysates with Western blot. Transfected HEK293T cells were treated with or without *N*-acetyl-l-leucyl-l-leucyl-l-norleucinal (ALLN) and Propranolol (Prop) at 24 or 48 h. HERG-WT, HERG-A561V, WT/A561V, HERG-L539fs/47, WT/L539fs/47, and Calnexin (CNX)/Calreticulin (CRT) protein expression and their interactions were detected by Western blot and immunoprecipitation. Each group with HERG currents (Ikr) were detected by Patch-clamp technique. Treated with ALLN, the expression of mature HERG protein and the CNX/CRT protein increased. The interaction of HERG-A561V and WT/A561V protein with the chaperone CNX/CRT increased significantly. The maximum peak currents and tail currents density increased by 70% and 73%, respectively, while maximal peak currents density (24%) and tail currents density (19%) were slight increased in WT-HERG cells. Treated with Prop, the expression and interaction of mature HERG and chaperones CNX/CRT had no difference in each group. The maximal currents and tail currents density were slight increased. CNX/CRT might play a crucial role in the trafficking-deficient process and degradation of HERG-A561V mutant protein, however they had no effect on L539fs/47 HERG due to protein transport deletion. ALLN can restore HERG-A561V mutant protein trafficking process and rescue the dominant negative suppression of WT-HERG.

## Introduction

The congenital long QT (LQT) syndrome is a heterogeneous genetic disease characterized by delayed cardiac repolarization, prolonged electrocardiographic QT intervals, the development of ventricular arrhythmias (Torsades de pointes) and sudden death, often in young healthy individuals, particularly children and teenagers [1–3]. LQT type 2 (LQT2) is the second most common type, caused by mutations in KCNH2 or the human ether-a-go-go-related gene (*hERG*) [4,5]. Defective trafficking of mutant channels to the cell membrane represent the most dominant mechanism of hERG channel dysfunction in LQT2 [6]. More than 500 hERG mutations having been identified, amongst which majority caused LQT2 due to HERG protein trafficking deficiency [7].

The endoplasmic reticulum (ER) is an important system for protein synthesis and cell processing, which is the strict quality control system to assemble protein correctly through the Golgi apparatus and reach the final site [8,9]. *HERG* mRNA synthesizes a protein monomer and core glycosylation (immature HERG protein showed the 135 kDa bands in Western blot) and core protein monomers into a dimer of four Ikr channels at the ER ribosome, which were transported to the Golgi complex of glycosylation (mature HERG protein showed the 155 kDa bands in Western blot). If gene mutation occurs, the protein is misfolded and trapped in the ER and then the disorder of transport occurs.

The defective trafficking proteins result in retention in the ER and fail to reach the plasma membrane [10]. At the same time, the unfolded protein response (UPR) was activated that increased the synthesis of chaperones’ proteins [11,12]. UPR consists of both translational and transcriptional regulations. Activating transcription factor 6 (ATF6) has been identified as a key regulator of transcriptional control in the mammalian UPR. Specifically, UPR activates the cleavage of ATF6 into its activated form, which then up-regulates the synthesis of ER chaperone proteins [13,14]. Misfolded and trafficking-deficient proteins retained in the ER are eventually degraded by a process termed as ER-associated degradation (ERAD). According to current models, ERAD substrates undergo retrotranslocation or dislocation from the ER to the cytosol, where they are degraded by the ubiquitination-proteasome pathway [15].

Molecular chaperone protein plays an important role in the biogenesis and quality control of glycolprotein. Calreticulin (CRT)/calnexin (CNX) is a key component of the chaperone protein for the folding of newly synthesized proteins/glycoproteins and quality control pathways in the ER [11,12,15]. We demonstrated that trafficking-deficient G572R-hERG and E637K-hERG mutant proteins activate ER stress pathways and target the proteasome for degradation. CNX and CRT play important roles in these processes [16]. Our preliminary test results showed that the HERG-A561V mutant protein was degraded by proteasome pathway. CNX/CRT, two chaperone proteins that arrested the mutant proteins in the ER and refolded them into a correct native conformation, might play crucial role in the trafficking-deficient and degradation processes of HERG-A561V mutant protein. L539fs/47 mutation encodes a truncated protein that does not contain N-linked glycans; L539fs/47 might lose the ability to bind CNX/CRT.

At present, the classic treatment of hereditary LQT is β blocker, but the specific mechanism is still not clear. The hereditary LQTS is still based on prevention, but cannot be cured. Therefore, the pathogenesis of hereditary LQTS is the key for its treatment.

## Materials and methods

### Antibodies and drugs

Rabbit polyclonal anti-hERG was purchased from Alomone (Alomone, Israel). Anti-CNX antibody was purchased from Santa Cruz Biotechnology (Santa Cruz, CA). Anti-CRT antibody was purchased from Abcam (Abcam, U.S.A.). Rabbit polyclonal anti-ATF6 was purchased from Active Motif (Active Motif, U.S.A.). The proteasome inhibitor of *N*-acetyl-l-leucyl-l-leucyl-l-norleucinal (ALLN) was purchased from Calbiochem, Propranolol (Prop) was purchased from Sigma (Sigma, U.S.A.).

### cDNA and cell culture

The WT-hERG was expressed by using the pCDNA3 vector (Invitrogen; Carlsbad, CA) [17]. A561V-HERG and L539fs/47-HERG cDNA were generated by site-directed mutagenesis of WT-hERG cDNA, and then subcloned into WT-hERG pCDNA3 vector at the BstEII/XhoI restriction sites. The functional effects of the A561V-HERG and L539fs/47-HERG were examined through transient expression in HEK-293 and U2SO cells. Transfections were carried out using Lipofectamine™ 2000 as described by the manufacturer (Invitrogen). The transfection successful proportion is 90%. HEK-293 and U2SO cells were cultured in Dulbecco’s modified Eagle’s medium supplemented with 10% FBS, maintained in a humidified 5% CO_2_ incubator at 37°C. HEK-293 and U2SO cells were not used beyond 30 passages.

### Drug intervention

After transfecting with pcDNA3-WT, pcDNA3-A561V, or pcDNA3-L539fs/47, respectively, HEK293 and U2SO cells were treated with or without 10 μM ALLN/10 μM Prop for 24 or 48 h. Each group of HERG currents (Ikr) were detected by using Patch-clamp technique. Part of the cell lysates were immunoblotted with anti-CNX/CRT and anti-hERG antibody for Western blotting. The other part of cell lysates was immunoprecipitated with anti-hERG and anti-CNX/CRT antibody. Then the cell lysates were subjected to SDS/PAGE and immunoblotted with anti-hERG antibody.

### Western blotting analysis

HEK293 cells expressing hERG in 100-mm diameter culture dishes were harvested for analysis after transient transfection for 48 h. Resuspended cells were washed with ice-cold PBS. Cell pellets were solubilized in ice-cold NP-40 buffer (20 mM Tris/HCl at pH 8, 137 mM NaCl, 10% glycerol, 1% NP-40, and 2 mM EDTA) plus a protease inhibitor PMSF (Sigma, U.S.A.) incubated on ice for 30 min, centrifuged at 15000 rpm for 15 min at 4°C to pellet detergent-insoluble cell debris and the supernatant was stored at −80°C. Proteins were separated on SDS/polyacrylamide gels, and then transferred on to PVDF membranes (Pierce Biotechnology). Membranes were blocked for 2 h at room temperature with blocking solution (5% non-fat dry milk powder and 0.2% Tween-20 in TBS). The membranes were incubated with rabbit polyclonal anti-hERG (1:200 dilution), rabbit polyclonal anti-ATF6 (1:200 dilution), mouse monoclonal anti-CNX (1:200 dilution) and anti-CRT (1:200 dilution) at 4°C overnight, respectively. After three washings with TBST, the membrane was blotted with an HRP-conjugated secondary antibody. Western blots were visualized with SuperSignal West Pico Chemiluminescent Substrate (Pierce Biotechnology) according to manufacturer’s instructions, and was performed using a Syngene Chemi-Genius imaging system (SynGene, U.K.). Image densities were quantitated using ImageJ. The experiment was repeated three times. Data are presented as mean ± S.E.M. Paired *t* test was used for statistical analysis, with *P*<0.05 considered significant.

### Coimmunoprecipitation of CNX/CRT and immature hERG

HEK293 cells were transiently transfected with pcDNA3-WT, pcDNA3-A561V, and pcDNA3-L539fs/47, then cell was incubated for 24 h with ALLN or Prop (10 μm), then lysed in 500 μl of immunoprecipitation buffer (50 mM Tris/HCl, pH 8.0, containing 150 mM NaCl, 1 mM CaCl_2_, and 1% Triton X-100) with protease inhibitors (100 μM PMSF, 1 μg/ml pepstatin A, 1 μg/ml leupeptin, 4 μl/ml aprotinin). After centrifugation at 13000 rpm for 15 min at 4°C, the cell lysates were precleared by incubation with protein G plus-agarose beads (Santa Cruz Biotechnology). The CNX-hERG/CRT-hERG complexes were immunoprecipitated by incubating with 2 μg antibody against CNX/CRT at 4°C overnight. The antigen–antibody complexes were isolated with protein G plus-agarose beads and washed with the immunoprecipitation buffer. The bound antigens were eluted from the protein G plus-agarose beads by 2× sample buffer and analyzed by immunoblotting with anti-hERG. Image densities were quantitated using ImageJ. The experiment was repeated three times. Data are presented as mean ± S.E.M. Paired *t* test was used for statistical analysis, with *P*<0.05 considered significant.

### Whole cell patch-clamp recordings

To examine the effect of ALLN and Prop on HERG-WT, HERG-A561V, HERG-WT/A561V by patch-clamp technique, HEK293 cells were harvested for 24 h (DNA plasmid only) after transfecting and superfusing with HEPES-buffered Tyrode solution. Membrane currents were recorded in whole-cell configuration using pipettes with tip resistance of 2–5 MΩ when filled with the internal solution as described in previous reports [7,18–20]. The electrodes were connected to an Axopatch 700B amplifier (Axon Instruments, Foster City, CA) and digitized at 2 kHz with analog-to-digital converter (DigiData1440A, Axon, U.S.A.). This experiment used pCLAMP version 10.3 software (Axon Instruments, California, U.S.A.) to edit the stimulus program, record the current, and analyze and measure the raw data. Excel and Origin7.5 software do the statistics and mapping of the original data, activate the current and the tail current, calculate the current density according to the cell capacitance, so as to eliminate the impact of cell size on the data. All data were expressed by mean and S.D. and paired *t* test was used before and after treatment. The difference of *P*<0.05 was significant.

## Results

### Effect of ALLN on the expression of hERG protein and molecular chaperones CNX/CRT in different cell models

To determine whether ALLN corrects the translocation of HERG-A561V mutant protein by inhibiting proteasome degradation pathway, the expression of HERG-WT, HERG-A561V, HERG-L539fs/47, HERG-WT/A561V, HERG-WT/L539fs/47 and CNX/CRT protein were detected by Western blot in transfected HEK293T cells treated with or without ALLN (10 μmol/l) after 24 or 48 h.

**Figure 1:**
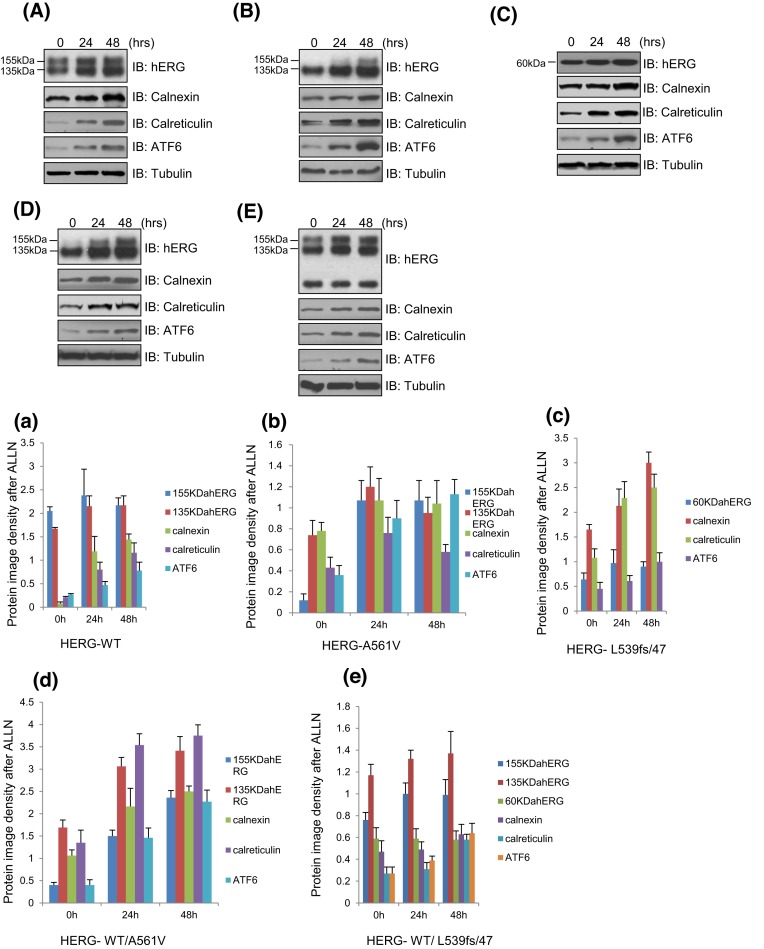
The overexpressed HERG-WT, HERG-A561V, HERG-L539fs/47, HERG-WT/A561V, HERG-WT/L539fs/47, and CNX/CRT protein were detected in transfected HEK293T cells treated with or without ALLN after 24 or 48 h (**A/a**) HERG-WT, (**B/b**) HERG-A561V, (**C/c**) HERG-L539fs/47, (**D/d**) HERG-WT/A561V, and (**E/e**) HERG-WT/L539fs/47.

As shown in the Figure 1A,B,D, after treatment with 10 μmol/l ALLN for 0, 24, or 48 h, protein levels of the hERG protein (135 and 155 kDa), CNX/CRT (90/60 kDa), ATF6 significantly increased in WT-Herg, HERG-A561V, HERG-WT/A561V, especially the mature HERG protein (155 kDa) in HERG-A561V and HERG/A561V (Figure 1A,B,D). In the Figure 1a,b,d, the protein levels were significantly increased after treating with 10 μmol/l ALLN for 48 h (*P*<0.05). While the protein intensity of HERG-L539fs/47, herg, CNX, CRT, and ATF6 were not increased significantly in HERG-WT/L539fs/47 cells treated with 10 μmol/l ALLN (Figure 1E/e). The protein intensity of CNX and CRT were increased significantly in HERG-L539fs/47 cells treated with 10 μmol/L ALLN, while herg and ATF6 were not (Figure 1C/c).

These results suggest that ALLN can correct the transport barrier of A561V mutant proteins, and the molecular chaperones may promote the degradation of HERG-A561V mutant proteins, but not L539fs/47.

### Effect of Prop on the expression of HERG protein and molecular chaperones CNX/CRT in different cell models

**Figure 2:**
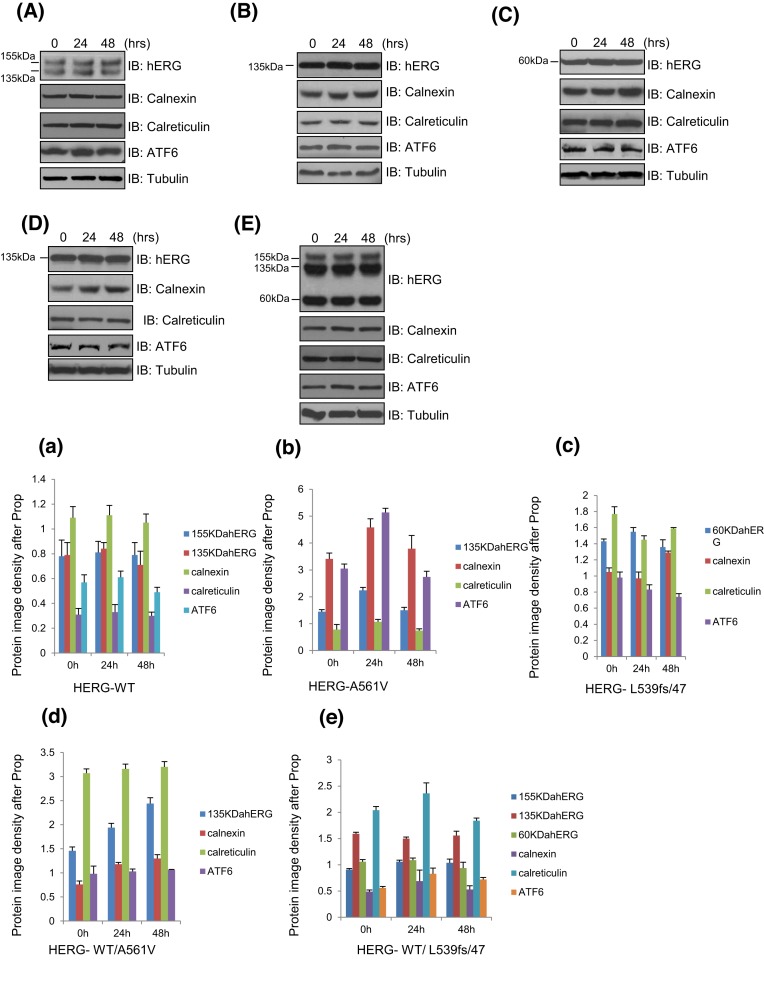
The overexpressed HERG-WT, HERG-A561V, HERG-L539fs/47, HERG-WT/A561V, HERG-WT/L539fs/47 and CNX/CRT protein were detected in transfected HEK293T cells treated with or without Prop after 24 or 48 h (**A**/a) HERG-WT, (**B/b**) HERG-A561V, (**C/c**) HERG-L539fs/47, (**D/d**) HERG-WT/A561V, and (**E/e**) HERG-WT/L539fs/47.

As shown in Figure 2A/a,B/b,D/d, after treating with 10 μM Prop for 0, 24, or 48 h, the protein levels of the hERG protein (135 and 155 kDa), CNX/CRT (90/60 kDa), ATF6 did not increase in WT-Herg, HERG-A561V, HERG-WT/A561Vcells, *P*>0.05. Meanwhile, the protein intensity of 60, 90, 135, and 155 kDa were not increased significantly in HERG-L539fs/47 or HERG-WT/L539fs/47 overexpressed cells treated with 10 μM Prop for 0, 24, or 48 h (Figure 2C/c,E/e). It was suggested that Prop could not correct the disorder of mutant protein transport, and molecular chaperone CNX/CRT did not play a role in this process.

### Effect of ALLN on the binding of HERG protein and molecular chaperone CNX/CRT in each cell model

**Figure 3:**
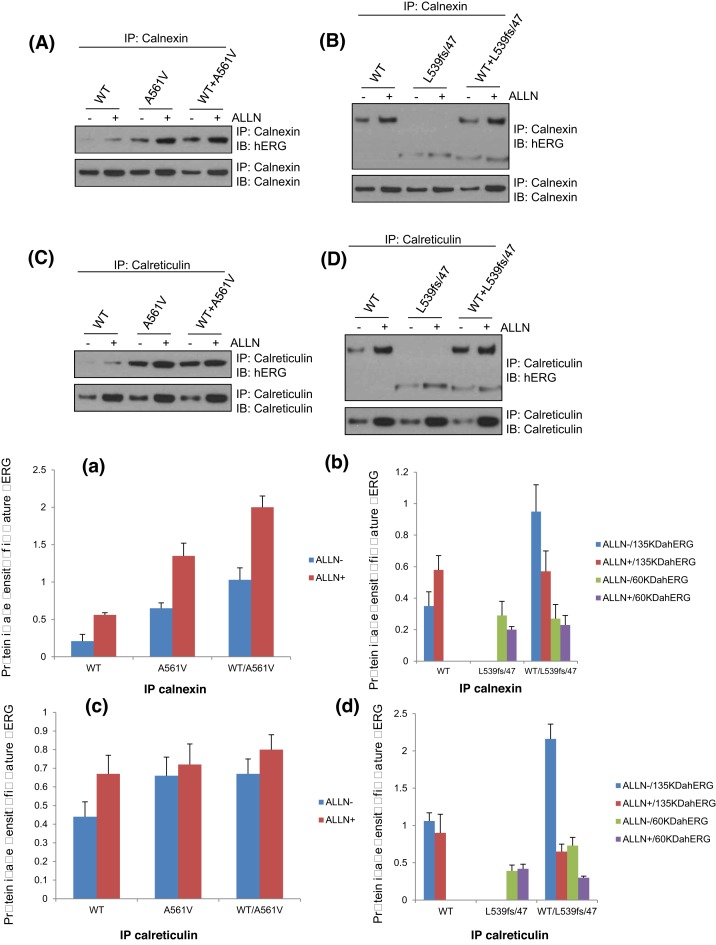
The interactions between the HERG-WT, HERG-A561V, HERG-L539fs/47, HERG-WT/A561V, HERG-WT/L539fs/47 protein with CNX(A/a,B/b)/CRT(C/c,D/d) were analyzed by immunoprecipitation after 24 h treatment with or without ALLN (10 μM)

To determine the role of chaperone CNX/CRT in the process of ALLN to correct the translocation of HERG-A561V mutant protein, the binding of HERG protein and molecular chaperone CNX/CRT in the HERG-WT, HERG- A561V, HERG-L539fs/47, HERG-WT/A561V, HERG-WT/L539fs/47 cell models were detected by immunoprecipitation after 24-h treatment with or without ALLN (10 μmol/l). The physical association of hERG channels with CNX and CRT was determined by immunoprecipitation with anti-CNX/anti-CRT antibody followed by Western blot with anti-hERG antibody. As shown in Figure 3A/a,C/c, the expression of CNX/CRT and its binding to HERG protein increased in the HERG-WT, HERG-A561V, HERG-WT/A561V cell models after 24 h of treatment with ALLN (10 μM), *P*<0.05. However, the impact of L539fs/47 is not significant (Figure 3B/b,D/d).

It indicated that the molecular chaperone CNX/CRT may affect the normal transport of mutant proteins though the ER retention and the degradation of proteasome pathway in HERG-A561V cells.

### Effect of Prop on the binding of HERG protein and molecular chaperone CNX/CRT in each cell model

**Figure 4:**
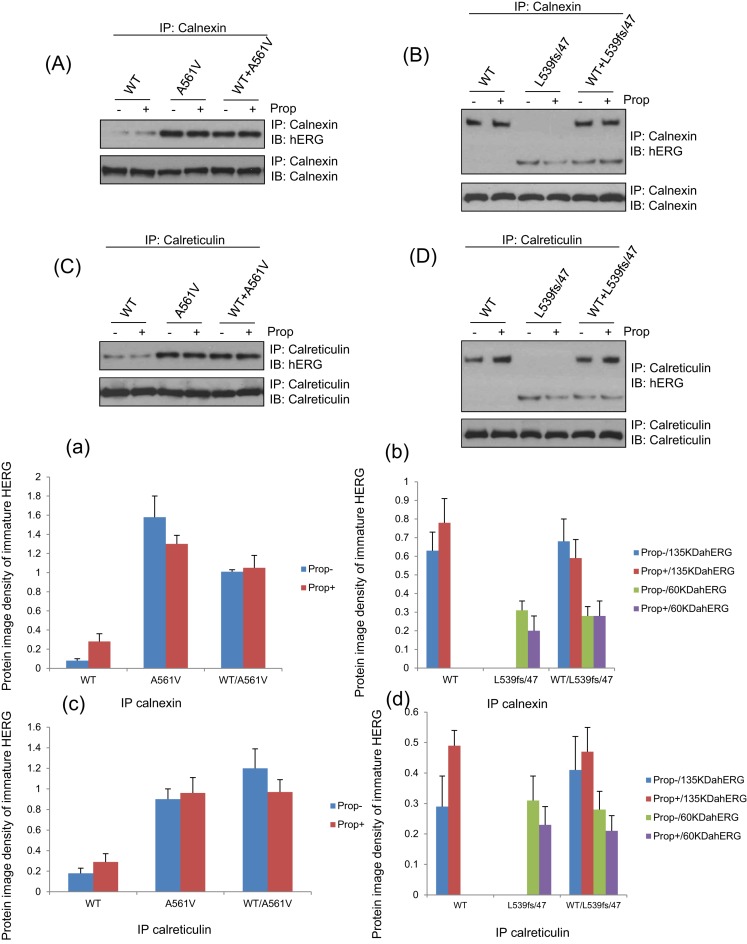
The interactions between the HERG-WT, HERG-A561V, HERG-L539fs/47, HERG-WT/A561V, HERG-WT/L539fs/47 protein with CNX(A/a,B/b)/CRT(C/c,D/d) were analyzed by immunoprecipitation after 24-h treatment with or without Prop (10 μM) after 24 h

As shown in the Figure 4A/a,B/b,C/c,D/d, after treating with Prop (10 μM) for 24 h, there was no significant change in the expression of CNX/CRT and their binding to HERG protein in HERG-WT, HERG-A561V, HERG-L539fs/47, HERG-WT/A561V, HERG-WT/L539fs/47 cells. The results showed that the localization of CNX, CRT and immature HERG did not change, *P*>0.05.

### Effect of ALLN on the activation of HERG and the change of tail current in each group

**Figure 5:**
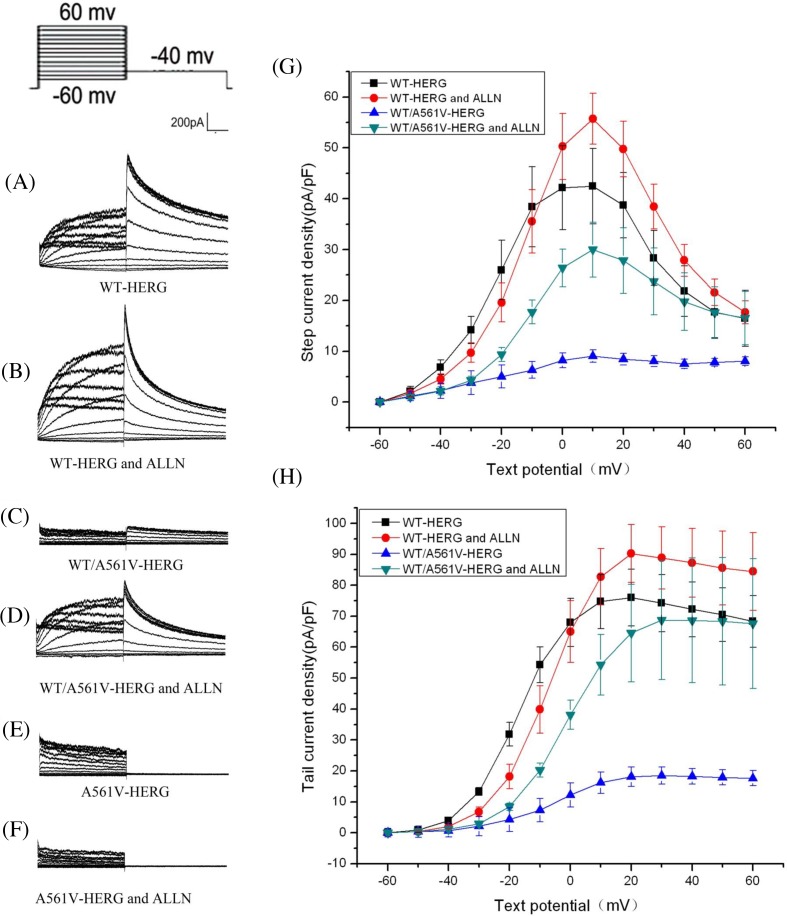
Effect of ALLN on the activation of HERG and the change of tail current in each group (**A–F**) represent the relationships for peak currents and tail currents amplitude of WT-HERG or A561V/HERG and A561V-HERG plasmids in presence or absence of ALLN. (**G**,**H**) represent the current density–voltage (I–V) relationships for peak currents and tail currents.

The results showed that ALLN impacts the peak currents of the forward and backward maximum activation current density at approximately +10 mV (before intervention: 42.46 ± 7.41 pA/pF, after intervention: 55.72 ± 5.04 pA/pF, increase: 31%, *n*=6, *P*>0.05) and tail currents amplitude (before intervention: 76.02 ± 9.2 pA/pF, after intervention: 90.25 ± 9.3 pA/pF, increase: 19%, *n*=6, *P*>0.05) increases slightly in HERG-WT, but there was no significant difference (Figure 5A,B,G,H). ALLN impacts the peak currents of the forward and backward maximum activation current density at approximately +10 mV in WT/A561V, the results were 9.05 ± 1.20 and 29.97 ± 5.4 pA/pF, increased by 231% (*n*=6, *P*<0.05), respectively. Similarly, the maximum tail current density of WT/A561V before and after ALLN interference was 18.46 ± 2.77 pA/pF and 68.78 ± 19 pA/pF, increased by 273% (*n*=6, *P*<0.05), which was significant (Figure 5C,D,G,H).

Compared with overexpressed WT/A561V cells without ALLN treatment, the maximum peak currents and tail currents density in cells treated with ALLN increased by 70% and 73%, respectively, while WT-HERG cells showed minimal increase in maximal peak currents density (24%) and tail currents density (19%). Furthermore, treatment with ALLN in HERG-A561V overexpressed cells had no effect on currents generation (Figure 5E,F).

Although ALLN corrected the abnormal transport of HERG-A561V mutation, the Ikr current was not detected when HERG-A561V was deleted before and after the intervention of patch-clamp. It indicated that although HERG-A561V transported the ER to the cell membrane, potassium ions still cannot pass channel due to their structure mutations of the channel pore region and their channel mouth.

### Effect of Prop on the activation of HERG and the change of tail current in each group

**Figure 6:**
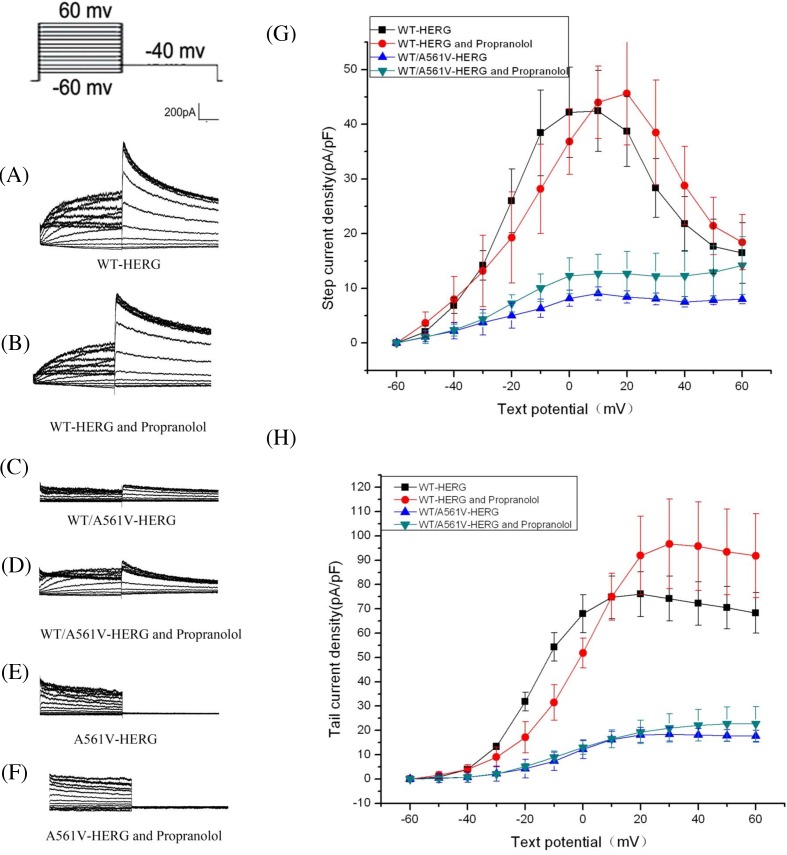
Effect of Prop on the activation of HERG and the change of tail current in each group (**A**–**F**) represent the relationships for peak currents and tail currents amplitude of WT-HERG or A561V/HERG and A561V-HERG plasmids in presence or absence of Prop. (**G**,**H**) represent the current density–voltage (I–V) relationships for peak currents and tail currents.

After treating with Prop, the maximal currents and tail currents density increase little. There was no significant difference in the maximum current density (before intervention: 42.46 ± 7.41 pA/pF, after intervention: 45.62 ± 9.4 pA/pF, increase: 7%, *n*=6, *P*>0.05) and the maximum current density of the tail current (before intervention: 76.02 ± 9.2 pA/pF, after intervention: 96.7 ± 18.45 pA/pF, increase: 21%, *n*=6, *P*>0.05) before and after interference with Prop in HERG-WT cells (Figure 6A,B,G,H). There was no significant difference in the maximum current density (before intervention: 9.05 ± 1.2 pA/pF, after intervention: 12.67 ± 3.58 pA/pF, increase: 28%, *n*=6, *P*>0.05) and the maximum current density of the tail current (before intervention: 18.46 ± 2.77 pA/pF, after intervention: 21 ± 5.8 pA/pF, increase: 12%, *n*=6, *P*>0.05) before and after interference with Prop in WT/A561V-HERG (Figure 6C,D,G,H). No current was detected in the HERG-A561V before and after interference with Prop (Figure 6E,F).

## Discussion

The initial hERG protein is translated in the ER. The nascent, immature protein undergoes a series of complex modifications before being transported to the cell membrane. In the ER, this quality control system always assists in protein folding and monitors its outcome, such as some misfolded proteins which are retained in the ER, performed as trafficking deficiency, and finally targetted for degradation [21,22]. Misfolded and unassembled proteins are retained in the ER and eventually degraded by a process termed as ERAD. According to the current models, ERAD substrates undergo retrotranslocation or dislocation from the ER to the cytosol, in which they are degraded by the proteasome [23].

However, the mechanism of ER quality control system, specifically for mutant protein trafficking-deficiency in ER, remains not fully clear. Previous studies have shown, amongst these modifications, chaperones play a crucial role in the assistance of protein folding and assembling, even in mutant protein trafficking process, while the proteasome plays important role in mutation protein degradation. Our previous study showed that the HERG-A561V and HERG-L539fs/47, two HERG mutation encoding proteins that have different trafficking processing, were degraded by the proteasome, and CNX/CRT and ATF6 play some role in their trafficking processes. While the L539fs/47 mutation encoded a truncated protein which had a location possession like WT-HERG and had no influence on WT-HERG trafficking (L539fs/47/WT-HERG).

To investigate the role of chaperones, CNX/CRT in the process of HERG-A561V trafficking defection, the present study worked on the HERG-A561V and L539fs/47 mutations, and detected the expression of HERG protein and molecular chaperone CNX/CRT in HERG-WT, HERG-A561V, HERG-L539fs/47, HERG-WT/A561V, HERG-WT/L539fs/47 cells. Data showed that with the longer treatment time of ALLN, mature HERG protein was significantly increased in HERG-A561V, HERG-WT/A561V cells, and the expression of molecular chaperone CNX/CRT also increased significantly. This demonstrated that ALLN can correct transport protein, and the molecular chaperone CNX/CRT may play a role in this process. Furthermore, the combination of the molecular chaperone CNX/CRT and HERG protein increased significantly with incubation of ALLN in HERG-A561V, HERG-WT/A561Vc cells, but not in HERG-L539fs/47, HERG-WT/L539fs/47 cells. This suggests that the molecular chaperone CNX/CRT may mediate the degradation of mutant HERG-A561V protein.

However, when the degradation pathway of HERG-A561V mutant protein was suppressed, it was transported to the cell membrane, but the similar results were not found in the HERG-G572R, HERG-L539fs/47, and HERG-E637K mutation sites. This may be caused by the protein in the ER transport quality monitoring system: different mutation, protein folding, and their corresponding retention and exit signal peptide exposure. With correct ER exit signal peptide, normal protein transport competed with its degradation. When degradation pathway was inhibited, the normal transport would be enhanced [24]. This study showed that mutant HERG-A561V protein cannot transport to cell membrane even without proteasome inhibitors. It may be caused by misfolded protein that was completely degraded by the proteasome degradation pathway. After incubation with ALLN, little HERG-A561V protein transport to cell membrane may be caused by ALLN’s effort to convert little HERG-A561V mutant protein into the right structure. But no current was detected due to channel mutant structure.

Prop is the most classical drug for the treatment to LQTS, and its mechanism is still unclear. The expression of HERG mature protein and molecular chaperone expression and the combination of the two did not significantly change in each group of cells after intervention with Prop 10 μM. Studies have shown that in heart failure dog model, the expression of CRT is increased, while the β blocker metoprolol to correct heart failure and regulate the normal balance of CRT regression [25]. In this experiment, the expression of CNX/CRT in each group was normal, and Prop did not correct the abnormal transfer of mutant protein, indicating that at least for the HERG-A561V and HERG-L539fs/47, it cannot achieve the purpose of treatment in LQTS.

ALLN corrects the transport barrier of mutant proteins HERG-A561V. To further define the functions of ALLN and Prop in each cell model, we used whole cell patch-clamp technique to detect Ikr current in each group after intervention with ALLN.

The Ikr current was not detected in HERG-A561V cells, which were transported from the ER to the cell membrane. But may be due to mutations in the channel pore region and the channel mouth, no potassium ions could pass its member channel.

The function of WT/A561V heterozygous channel is not completely defective. It is only retained in the ER due to structural abnormalities. When the cell membrane can be transported normally, it can play a certain compensatory function. However, no current was detected in the HERG-A561V before and after Prop’s interference. The cytological experiments showed that the effect of Prop on the wild-type HERG channel was depressed [26]. Other studies have shown that the treatment of hereditary LQTS with Prop may be related to the inhibition of sodium, potassium, and calcium channels, and reduce the dispersion of repolarization. The specific mechanism needs further study.

The present study shows that CNX/CRT, two chaperone proteins that arrested the mutant proteins in the ER and re-folded them into a correct native conformation, might play crucial role in the trafficking-deficient process and degradation of HERG-A561V mutant protein. ALLN can restore HERG-A561V mutant protein into trafficking process, also rescue the dominant negative suppression of WT-HERG. This potential approach may provide some reference in other clinically relevant protein trafficking disorders. Moreover, CNX/CRT might be involved in this procession. However, Prop cant rescue the trafficking deficiency of HERG-A561V, neither rescue the dominant negative suppression of WT-HERG. Also, CNX/CRT did not involve in this procession.

## Abbreviations

ALLN: *N*-acetyl-l-leucyl-l-leucyl-l-norleucinal
ATF6: activating transcription factor 6
CNX: calnexin
CRT: calreticulin
ER: endoplasmic reticulum
ERAD: ER-associated degradation
HERG(KCNH2): human-ether-a-go-go-related gene
Ikr: rapidly activating delayed rectifier K-current
LQT: long QT
LQTS: long QT syndrome
LQT2: LQT type 2
Prop: propranolol
QT: QT interval
UPR: unfolded protein response
